# Supplementary material for: Gaps in the HIV diagnosis and care cascade for migrants in Australia, 2013–2017: A cross-sectional study
Source: PLoS Med. 2020 Mar 10;17(3):e1003044. doi: 10.1371/journal.pmed.1003044 (PMC7064172; doi:10.1371/journal.pmed.1003044)
Supplement: S1 Text — (DOCX) [file pmed.1003044.s002.docx]

**S1 Text_Concept Sheet**

**Concept sheet**

Australian Collaboration for Coordinated Enhanced Sentinel Surveillance (ACCESS)

| **1) Submission date** | | |
| --- | --- | --- |
| 8/05/2017 | | |
| **2) Contact name and email** | | |
| Tafireyi Marukutira: [tafireyi.marukutira@burnet.edu.au](mailto:tafireyi.marukutira@burnet.edu.au) | | |
| **3) Study title** | | |
| HIV treatment cascade for migrants in Australia | | |
| **4) Proposed authors** | | |
| Tafireyi Marukutira, Praveena Gunaratnam, Richard Gray, Graham Brown, Roanna Lobo, Carol El-Hayek, Mark Stoove, Rebecca Guy, Margaret Hellard. On behalf of the National Cascade Reference Group and ACCESS | | |
| **5) Where are the data to be analysed?** *(For data analysis outside of the Kirby or Burnet Institutes, please contact the network coordinator)* | | |
| Burnet Institute | | |
| **6) Background and references** | | |
| Migrant populations from sub-Saharan Africa and South-East Asia accounted for 17% (4,364/25,313) of people living with HIV in Australia in 2015 despite only making up 5% of the total population aged 15 years and over.^1^ Sub-Saharan Africa and South East Asian individuals with HIV are also more likely to remain undiagnosed or present late irrespective of whether they acquired the infection through heterosexual or male-to-male sex. Between 2010 and 2015, the proportion with late diagnoses was highest in people born in South-East Asia (48%) and sub-Saharan Africa (46%).^1^  While the HIV epidemic in Australia is driven by gay and bisexual men (GBM) who accounted for 68% of all notifications in 2015, while 20% was due to heterosexual transmission. Of that subgroup, people born in countries with HIV prevalence above 1% (high prevalence) accounted for 19% of heterosexual transmissions while 17% had partners in high HIV prevalence countries.^1^ Migrants also form part of the culturally and linguistically diverse (CALD) groups in Australia who face numerous linguistic, cultural and financial barriers to health care.^2^ A migrant is defined as “a person who moves from one place to another in order to find work or better living conditions” (<http://www.dictionary.com>) and defined as an individual born overseas in this study. CALD people are generally migrants and defined as those people born overseas, in countries other than those classified by the Australian Bureau of Statistics (ABS) as “main English speaking countries” (Canada, the Republic of Ireland, New Zealand, South Africa, the United Kingdom and the United States of America. Australia receives migrants from all over the word including the high HIV prevalent regions such as South-East Asia, sub-Saharan Africa and Eastern Africa.  The United Nations Joint Program for AIDS (UNAIDS) adopted the ambitious 90-90-90 targets by 2020 in the hope of seeing the end of the AIDS epidemic by 2030.^3^ The targets entails 90% of estimated people living with HIV (PLWHIV) diagnosed, 90% of those diagnosed on antiretroviral therapy (ART) and 90% of those on ART with viral suppression.  In 2015, Australia had 90% of the estimated people living with HIV diagnosed, 75% on ART and 92% with viral suppression.^1^ Despite nearly achieving the 90:90:90 target no jurisdiction in Australia has observed a long-term decrease trend in new HIV infections over the past ten years^1^.Reaching the 90-90-90 targets will take inclusion of all key and vulnerable population groups in Australia. One of the vulnerable groups is the migrant community (CALD).  The national HIV treatment cascade has been described including that of GBM; however treatment cascades of other sub-populations have not been elucidated. As we work towards the 90-90-90 targets, but more importantly as we aim to reduce the number of new cases of HIV in Australia there is need to understand and identify gaps in the treatment cascade in other key and vulnerable population groups.  1. The Kirby Institute. HIV, viral hepatitis and sexually transmissible infections in Australia: Annual Surveillance Report. The Kirby Institute; 2016.  2. Mengesha ZB, Dune T, Perz J. Culturally and linguistically diverse women. Sex Health. 2016.  3. UNAIDS. 90-90-90 An ambitious treatment target to help end the AIDS epidemic. 2014. | | |
| **7) Objectives** | | |
| 1. To develop an HIV testing and treatment cascade for migrants in Australia 2. To compare the HIV testing and treatment cascade for migrants with other available cascades (e.g. national, GBM and other) 3. To identify steps in the cascade where there are gaps which will inform HIV care and prevention initiatives for migrant populations in Australia. | | |
| **8) Study population** | | |
| People living and diagnosed with HIV in Australia as of 2015 | | |
| **9) Inclusion and exclusion criteria** | | |
| Date range: 01/01/2015- 31/12/15 |  | |
| Age range | All ages | |
| Sex/gender | 🗹Male 🗹 Female 🗹 Intersex | |
| State/regions | All states and territories | |
| Priority populations | Gay/bisexual  Sex workers  Injecting drug users 🗹 All patients | |
| Others (specify) | Migrants/CALD | |
| **10) Data variables required** *(Refer to ACCESS data dictionary for more information)* | | |
| *Consultation data*:  🗹Clinic name  🗹Visit date  Visit type  Attendance reason  *Patient data*:  🗹Patient age  🗹Patient gender  ATSI status  🗹  🗹Patient postcode  🗹Country of birth  Traveller status & year of arrival in Australia  🗹Preferred language  *Treatment data:*  🗹Antiretroviral treatments  Other treatments: Click here to enter text. | | *Behavioural data:*  🗹Condom use  🗹Drug use  🗹Sexual partner information  🗹Sex work information  *Tests, results & diagnoses:*  Chlamydia  Gonorrhoea  Syphilis  🗹HIV  🗹HIV management (viral load; CD4 cell)  Hepatitis A  Vaccination information  Hepatitis B  Vaccination information  Hepatitis C  🗹Other(s): Transmission exposure, gender of sexual partners in last 12 months, record of death. |
| **11) Proposed analysis**   \|  \| **Variable** \| **Definition** \| **Data source** \| \| --- \| --- \| --- \| --- \| \|  \| Estimated number of migrants living with HIV (MLHIV) \| Estimated number of migrants living with HIV in the overall population (diagnosed, deaths and undiagnosed). Stratified by country of birth/region/language and exposure category. \| **Diagnosed**: Notifications from National HIV Registry.  Estimate of number undiagnosed.  **Deaths**: To estimate the number of deaths up to 2003 we will use a linkage study conducted between Australia's National Death Index and the National HIV Registry for cases to the end of 2003.For data from 2003 onward we will use annual mortality rates from the Australian HIV Observational Database (AHOD) with 95% confidence intervals.  **Undiagnosed**: estimate the proportion of PLHIV who are undiagnosed, using the European Centre for Disease Control HIV Modelling Tool (<http://ecdc.europa.eu/en/healthtopics/aids/pages/hiv-modelling-tool.aspx>).This model uses annual HIV, AIDS notifications and the annual number of people with a CD4 at diagnosis of >500, 350-499, 200-349, and <200 cells/μL to estimate the number of new infections and number of undiagnosed PLHIV. The estimate will be weighted according to the CD4 count in different exposure groups of migrants living with HIV. \| \|  \| Number of MLHIV diagnosed \| Number of newly diagnosed or known MLHIV as of 2015. \| Notifications from National HIV Registry \| \|  \| Number of MLHIV diagnosed linked to care \| Number of MLHIV diagnosed who underwent a CD4 or VL test within 3 months of diagnosis OR entry into the country OR clinic visit for an HIV related visit \| *ACCESS data. Also - Victorian sentinel data may be able to help for the past five years or so. \| \|  \| Number of MLHIV diagnosed retained in care \| Number of MLHIV who received at least one CD4 or VL test or attended clinic within the last 12 months (Jan-Dec 2015) \| *ACCESS data. Also check with AHOD and double check with WA or other jurisdiction for any state specific data. \| \|  \| Number of MLHIV diagnosed taking ART \| Number of MLHIV diagnosed who received antiretroviral drugs at some stage during last 12 months (in 2015) \| *ACCESS data. (?Sentinel data) \| \|  \| Number of MLHIV with suppressed virus \| Number of MLHIV taking ART in the last 12 months who had a VL less than 200 copies/ml at last test \| *ACCESS clinical/lab data \| | | |
| *ACCESS Data will be from ALL facilities. Depending on the completion rate of country of birth (COB), a sub-analysis using networks that have at least 60% completion rate of COB will be conducted.  Cascade:   - **Proportion of MLHIV diagnosed (1^st^ 90)**   Numerator: Number of newly diagnosed or known MLHIV  Denominator: Estimated number of migrants living with HIV in the overall population   - **Proportion of MLHIV diagnosed on ART (2^nd^ 90)**   Numerator: Number of MLHIV diagnosed taking ART  Denominator: Number of newly diagnosed or known MLHIV   - **Proportion of MLHIV on ART with viral suppression (3^rd^ 90)**   Numerator: Number of MLHIV with suppressed virus  Denominator: Number of MLHIV diagnosed taking ART   - **Overall cascade**   Numerator: Number of MLHIV with suppressed virus  Denominator: Estimated number of migrants living with HIV in the overall population | | |
| **12) Potential limitations** | | |
| Completeness of data especially documentation of country of birth. Depending on the initial data, analysis may be limited facilities with at least 60% completeness of country of birth. | | |
| **13) Significance** | | |
| Reaching and sustaining the UNAIDS 90-90-90 targets for HIV epidemic control requires that all potential gaps are identified and addressed. The HIV testing and treatment cascade for migrants will identify gaps in the cascade of care for migrants and make recommendations where there are gaps. | | |
| **14) How will the information be used?** | | |
| Peer reviewed publications, conference presentations. All services will be acknowledged in the manuscript. | | |
|  | | |
| To be completed by ACCESS coordinator  *Date received:*  *Date approved:* | | |

Save using the following convention: [First author surname] Short title DD.MM.YYYY.docx
