# Supplementary material for: Gaps in the HIV diagnosis and care cascade for migrants in Australia, 2013–2017: A cross-sectional study
Source: PLoS Med. 2020 Mar 10;17(3):e1003044. doi: 10.1371/journal.pmed.1003044 (PMC7064172; doi:10.1371/journal.pmed.1003044)
Supplement: S1 Table — PLHIV, people living with HIV. (DOCX) [file pmed.1003044.s003.docx]

**S1 Table: Estimated people living with HIV (PLHIV) and proportion diagnosed by gender, MSM and migration status (2013-2017)**

|  | **2013**  **N (95%CI)** | **2014**  **N (95%CI)** | **2015**  **N (95%CI)** | **2016**  **N (95%CI)** | **2017**  **N (95%CI)** |
| --- | --- | --- | --- | --- | --- |
| **PLHIV** | 15,360 (13,370-17,020) | 15,990 (13,880-17,780) | 16500 (14,220-18,420) | 17160 (14,710-19,210) | 17,760 (15,110-19,980) |
| *Male* | 13,853 | 14,417 | 14,853 | 15,437 | 15,961 |
| *Female* | 1,505 | 1,572 | 1,647 | 1,724 | 1,796 |
| **Migrants** | 5,399 | 5,666 | 5,942 | 6,263 | 6,571 |
| *Male* | 4,487 | 4,709 | 4,933 | 5,200 | 5,471 |
| *Female* | 909 | 963 | 1,013 | 1,071 | 1,110 |
| **Non-migrant** | 10,011 | 10,384 | 10,612 | 10,917 | 11,196 |
| *Male* | 9,462 | 9,822 | 10,037 | 10,330 | 10,587 |
| *Female* | 520 | 533 | 557 | 577 | 610 |
| **Male-to-male HIV exposure** | 12,220 | 12,706 | 13,086 | 13,534 | 13,945 |
| *Migrant* | 3,587 | 3,772 | 3,989 | 4,218 | 4,462 |
| *Non-migrant* | 8,633 | 8,934 | 9,097 | 9,316 | 9,483 |
| **Born in SEA** | 1,214 | 1,316 | 1,430 | 1,582 | 1,700 |
| **Born in SSA** | 674 | 711 | 732 | 764 | 787 |
| **RHCA eligible** | 1,322 | 1,349 | 1,378 | 1,401 | 1,415 |
| **RHCA not eligible** | 4,077 | 4,317 | 4,564 | 4,862 | 5,156 |
| **Diagnosed** | 13,708 | 14,331 | 14,833 | 15,458 | 16,034 |
| *Male* | 12,394 | 12,,931 | 13,348 | 13,903 | 14,401 |
| *Female* | 1,305 | 1,375 | 1450 | 1,525 | ,1595 |
| **Migrants** | 4,496 | 4,755 | 5,022 | 5,326 | 5,618 |
| *Male* | 3,727 | 3,933 | 4,143 | 4,390 | 4,640 |
| *Female* | 769 | 823 | 871 | 926 | 9,63 |
| **Non-migrant** | 9,340 | 9,700 | 9,925 | 10,222 | 10,495 |
| *Male* | 8,826 | 9,170 | 9,376 | 9,653 | 9,896 |
| *Female* | 487 | 498 | 520 | 536 | 563 |
| **Male-to-male HIV exposure** | 11,005 | 11,466 | 11,844 | 12,280 | 12,677 |
| *Migrant* | 2,994 | 3,154 | 3,344 | 3,544 | 3,756 |
| *Non-migrant* | 8,182 | 8,486 | 8,662 | 8,891 | 9,074 |
| **Born in SEA** | 845 | 925 | 1,014 | 1,135 | 1,225 |
| **Born in SSA** | 558 | 603 | 633 | 672 | 703 |
| **RHCA eligible** | 1,241 | 1,275 | 1,311 | 1,338 | 1,358 |
| **RHCA not eligible** | 3,255 | 3,480 | 3,711 | 3,988 | 4,260 |

PLHIV: people living with HIV; MSM: gay, bisexual, and other men who have sex with men; RHCA: Reciprocal healthcare agreement with Australia (eligible countries are. Belgium, Finland, Italy, Malta, Netherlands, New Zealand, Norway, Ireland, Slovenia, Sweden, and United Kingdom); Sub-Saharan Africa: includes Southern, Eastern, West, and Middle Africa. IRR: Incidence rate ration (Poisson regression); CI confidence interval; ART: antiretroviral therapy; VL: viral load.
