# Supplementary material for: Gaps in the HIV diagnosis and care cascade for migrants in Australia, 2013–2017: A cross-sectional study
Source: PLoS Med. 2020 Mar 10;17(3):e1003044. doi: 10.1371/journal.pmed.1003044 (PMC7064172; doi:10.1371/journal.pmed.1003044)
Supplement: S2 Table — ART, antiretroviral therapy; PLHIV, people living with HIV. (DOCX) [file pmed.1003044.s004.docx]

**S2 Table: Number retained in care and viral load done if on ART, by gender, MSM and Migration Status (2013-2017)**

|  | **2013** | **2014** | **2015** | **2016** | **2017** |
| --- | --- | --- | --- | --- | --- |
| **Retained** | **8,639** | **9,042** | 9,333 | 9,607 | 9,391 |
| *Male* | 7,490 | 7,812 | 8,061 | 8,231 | 8,015 |
| *Female* | 482 | 503 | 524 | 542 | 531 |
| *Missing sex* | 661 | 719 | 741 | 826 | 827 |
| **Missing country of birth** | 3,772 | 3,718 | 3,784 | 3,697 | 3,574 |
| **Migrants** | 1,763 | 1,967 | 2,086 | 2,300 | 2,408 |
| *Male* | 1,264 | 1,411 | 1,505 | 1,657 | 1,742 |
| *Female* | 213 | 234 | 240 | 254 | 262 |
| *missing* |  |  |  |  |  |
| **Non-migrant** | 3,104 | 3,357 | 3,463 | 3,610 | 3,409 |
| *Male* | 2,563 | 2,802 | 2,894 | 3,013 | 2,829 |
| *Female* | 178 | 169 | 185 | 182 | 169 |
| *missing* |  |  |  |  |  |
| **Male-to-male HIV exposure** | 3,481 | 3,818 | 3,979 | 4,213 | 4,110 |
| *Migrant* | 1,119 | 1,267 | 1,349 | 1,497 | 1,558 |
| *Non-migrant* | 2,362 | 2,551 | 2,630 | 2,716 | 2,552 |
| *Missing cob* |  |  |  |  |  |
| **Born in SEA** | 349 | 410 | 448 | 528 | 556 |
| **Born in SSA** | 222 | 239 | 238 | 258 | 267 |
| **RHCA eligible** | 510 | 540 | 541 | 550 | 535 |
| **RHCA not eligible** | 1,253 | 1,427 | 1,545 | 1,750 | 1,873 |
| **On ART** | **6,183** | **6,672** | **7,196** | **8,254** | **8,489** |
| *Male* | 5,269 | 5,682 | 6,129 | 7,055 | 7,267 |
| *Female* | 320 | 338 | 374 | 412 | 414 |
| *Missing sex* | 591 | 649 | 691 | 782 | 798 |
| **Missing country of birth** | 2,647 | 2,704 | 2,844 | 3,368 | 3,376 |
| **Migrants** | 1,247 | 1,420 | 1,619 | 1,857 | 2,058 |
| *Male* | 846 | 968 | 1,124 | 1,301 | 1,469 |
| *Female* | 148 | 162 | 179 | 196 | 208 |
| **Non-migrant** | 2,289 | 2,548 | 2,733 | 3,029 | 3,055 |
| *Male* | 1,841 | 2,084 | 2,242 | 2,499 | 2,535 |
| *Female* | 120 | 115 | 132 | 130 | 119 |
| **Male-to-male HIV exposure** | 2,468 | 2,797 | 3,110 | 3,513 | 3,690 |
| *Migrant* | 755 | 885 | 1,033 | 1,211 | 1,352 |
| *Non-migrant* | 1,713 | 1,912 | 2,077 | 2,302 | 2,338 |
| *Missing cob* |  |  |  |  |  |
| **Born in SEA** | 264 | 314 | 370 | 437 | 486 |
| **Born in SSA** | 177 | 199 | 209 | 231 | 247 |
| **RHCA eligible** | 357 | 395 | 416 | 442 | 458 |
| **RHCA not eligible** | 890 | 1,025 | 1,203 | 1,415 | 1,600 |
| **Viral load done** | **4,783** | **5,318** | **5,701** | **6,650** | **6,853** |
| *Male* | 4,017 | 4,478 | 4,791 | 5,617 | 5,736 |
| *Female* | 229 | 254 | 284 | 305 | 307 |
| *Missing sex* | 537 | 586 | 626 | 728 | 810 |
| **Missing country of birth** | 1,867 | 2,007 | 2,064 | 2,567 | 2,560 |
| **Migrants** | 1,052 | 1,234 | 1,375 | 1,577 | 1,744 |
| *Male* | 710 | 843 | 954 | 1,099 | 1,215 |
| *Female* | 110 | 132 | 140 | 154 | 154 |
| **Non-migrant** | 1,864 | 2,077 | 2,262 | 2,506 | 2,549 |
| *Male* | 1,479 | 1,674 | 1,827 | 2,034 | 2,041 |
| *Female* | 90 | 87 | 107 | 95 | 99 |
| **Male-to-male HIV exposure** | 3,868 | 4,306 | 4,614 | 5,444 | 5,587 |
| *Migrant* | 664 | 787 | 898 | 1,046 | 1,172 |
| *Non-migrant* | 1,407 | 1,583 | 1,734 | 1,943 | 1,965 |
| *Missing cob* |  |  |  |  |  |
| **Born in SEA** | 236 | 286 | 339 | 389 | 432 |
| **Born in SSA** | 149 | 169 | 176 | 195 | 198 |
| **RHCA eligible** | 300 | 337 | 345 | 368 | 385 |
| **RHCA not eligible** | 752 | 897 | 1,030 | 1,209 | 1,359 |
| **Viral suppression (all on ART)** | **4,237** | **4,832** | **5,263** | **6,293** | **6,528** |
| *Male* | 3,550 | 4,070 | 4,420 | 5,322 | 5,470 |
| *Female* | 193 | 220 | 248 | 279 | 278 |
| *Missing sex* | 494 | 542 | 595 | 692 | 780 |
| **Missing country of birth** | 1686 | 1871 | 1944 | 2462 | 2465 |
| **Migrants** | 910 | 1,099 | 1232 | 1,470 | 1,619 |
| *Male* | 602 | 739 | 843 | 1,023 | 1,121 |
| *Female* | 93 | 116 | 121 | 140 | 139 |
| **Non-migrant** | 1,624 | 1,846 | 2,073 | 2,357 | 2,439 |
| *Male* | 1,279 | 1,486 | 1,667 | 1,908 | 1,956 |
| *Female* | 75 | 73 | 92 | 87 | 87 |
| **Male-to-male HIV exposure** | 3,442 | 3,941 | 4,262 | 5,179 | 5,346 |
| *Migrant* | 574 | 697 | 793 | 976 | 1,091 |
| *Non-migrant* | 1,223 | 1,421 | 1,586 | 1,839 | 1,889 |
| **Born in SEA** | 195 | 257 | 295 | 359 | 402 |
| **Born in SSA** | 130 | 154 | 162 | 179 | 181 |
| **RHCA eligible** | 269 | 307 | 314 | 353 | 366 |
| **RHCA not eligible** | 641 | 792 | 918 | 1,117 | 1,253 |

***PLHIV****: people living with HIV;* ***MSM****: gay, bisexual, and other men who have sex with men;* ***RHCA****: Reciprocal healthcare agreement with Australia (eligible countries are. Belgium, Finland, Italy, Malta, Netherlands, New Zealand, Norway, Ireland, Slovenia, Sweden, and United Kingdom);* ***Sub-Saharan Africa****: includes Southern, Eastern, West, and Middle Africa.*

***IRR****: Incidence rate ration (Poisson regression****); CI*** *confidence interval;* ***ART****: antiretroviral therapy;* ***VL****: viral load*
