# Supplementary material for: Gaps in the HIV diagnosis and care cascade for migrants in Australia, 2013–2017: A cross-sectional study
Source: PLoS Med. 2020 Mar 10;17(3):e1003044. doi: 10.1371/journal.pmed.1003044 (PMC7064172; doi:10.1371/journal.pmed.1003044)
Supplement: Attachment — Submitted filename: Responses to reviewers_FINAL.docx [file pmed.1003044.s005.docx]

**RESPONSES TO REVIEWERS**

**Requests from the editors:**

**Comment:** Noting referee 1's comments, PLOS Medicine policy is that significance tests should be performed and reported with 95% CI and p values where appropriate.

***Response:*** *Thank you and duly noted. We have addressed Reviewer #1 comments making reference to the PLOS Medicine policy on significance tests.*

**Comment:** Please adapt the title so that the segment after the colon consists predominantly of a study descriptor, e.g., "HIV Diagnosis and Care Cascade for Migrants in Australia: a cross-sectional study", and include study date(s).

***Response:*** *Thank you, the title has been edited to: Gaps in the HIV diagnosis and care cascade for migrants in Australia: a cross-sectional study (2013-2017)*

**Comment:** We ask you to combine the "methods" and "findings" subsections of your abstract. Please add a new final sentence to the new combined subsection to summarize the study's main limitations.

**Response:** The following limitation was added: *“The major limitation of our study was a high proportion (38% in 2017) of individuals missing a country of birth entry thereby limiting migrant status categorization”.*

**Comment:** Please add summary demographic and country or region of origin details for study participants to abstract and main text.

***Response:*** *Thank you for highlighting this. Summary demographic and region of origin details have been added in the abstract and main text.*

**Comment:** After the abstract, we will need to ask you to add a new and accessible "author summary" section in non-identical prose. You may find it helpful to consult one or two recently published research papers in PLOS Medicine to get a sense of the preferred style.

***Response:*** *The “Author Summary” has been added with subheadings: 1. Why was this study done? 2) What did the researchers do and find? and 3) What does these findings mean?*

**Comment:** Early in the methods section, please state whether or not the study had a protocol or prespecified analysis plan, and if so attach the document(s) as a supplementary file (referred to in the text). Please highlight analyses that were not prespecified.

***Response:*** *A pre-specified analysis plan was used attached as supplementary information S1*

**Comment:** Please avoid claims of "the first", and where used (e.g. currently at the start of the Discussion section) please add "to our knowledge" or similar.

***Response:*** *Thank you for highlighting this. The first sentence has been edited to: “To the best of our knowledge, this is the first analysis that examined and compared HIV diagnosis and the care cascade in migrants in Australia”.*

**Comment:** The first paragraph of the discussion should summarize the study's findings, with elements of discussion being restricted to subsequent paragraphs.

***Response:*** *The first paragraph was edited to highlight the study’s major findings as:*

*“Our results suggest that while gaps in the overall HIV diagnosis and care cascade in NSW and VIC decreased between 2013 and 2017, there were notable and sustained gaps in migrant subpopulations. Overall, migrants had lower HIV diagnosis and retention across the HIV care cascade than non-migrants, especially the proportion diagnosed and on ART (first and second 90). Migrants reporting male-to-male HIV exposure, born in SEA and SSA, ineligible for subsidized healthcare through RHCA and females had lower HIV diagnosis and retention across the care cascade than non-migrants. There was a particularly low proportion of PLHIV born in SEA diagnosed (72% in 2017). As we approach 2020, the first Fast-Track target year, we estimated that in 2017, 70% and 65% of migrants estimated to be living with HIV in NSW and VIC were on ART and virally suppressed respectively. This was lower than 80% and 77% respectively in non-migrants estimated to be living with HIV.”*

**Comment:** Please ensure that all references meet journal format. For example, "and" should be removed from the author list for reference 4, and journal names should be abbreviated as appropriate.

***Response:*** *References have been edited and formatted as per journal requirements. Journal names have been abbreviated as appropriate.*

**Comment:** Please correct "report" at line 410.

***Response:*** *Thank you, the correction has been made.*

**Comment:** Please add a completed checklist for the most appropriate reporting guideline, which might be STROBE or RECORD, as a supplementary document (referred to in the methods section). In the checklist, please refer to individual items by section (e.g., "Methods") and paragraph number rather than by page or line numbers, as the latter generally change in the event of publication.

***Response:*** *The STROBE Statement for observational studies has been added as S2 STROBE Checklist.*

***** Reviewer #1:**

**Comment:** This is an important study and shows important information and I think the authors have done a nice job with the study. There are a few places where I think the reporting is unclear and needs some revising and possibly the methods, but I think these changes are not overly major. Below are specific comments for the authors. I do want to commend the authors on the reporting of precisely null results! This is important to know, but often gets confused with non-significance. I would agree your findings are null even though you are near significance! My only quibble would be to ask you not to use significance testing at all and remove reference to "with the significance level set at p<0·05" in the methods and remove the p-values from the results.

***Response:*** *Thank you for highlighting this evolving subject on significance testing. We agree that sometimes there is over emphasise on significance levels and in this manuscript we report both 95% CI and p-values were appropriate. We also want to acknowledge that the journal, PLOS Medicine’s policy is that significance tests should be performed and reported with 95% CI and p values where appropriate.*

**Comments:** In the methods, can you clarify this statement, "To estimate the number of people living with diagnosed HIV (PLDHIV) we used New South Wales (NSW) and Victorian (VIC) data from the Australian National HIV Registry, enhanced by surveillance among people recently diagnosed with HIV to improve migration estimates." What does it mean to be "enhanced"?

***Response:*** *Thank you for the comment. In addition to the data in the Australian National HIV Registry the health departments in each Australian jurisdiction engage in follow-up activities to fill in missing data and determine what happens to people after they have been diagnosed. For example, in the last few years NSW has conducted a 6-monthly follow-up of people diagnosed with HIV to determine if they are still in Australia, in care, and taking ART. These data were used in our calculations. We have edited this sentence to clarify.*

**Comment:** You note that "The ACCESS network collects demographic, testing, diagnosis and treatment data to monitor the sexual health of high‑ risk populations. Compared to other states, NSW and VIC have the highest coverage in the ACCESS database and they comprise the highest proportion of PLHIV and migrants in Australia [6, 17]." But it is important to state here how high the coverage is. Is this the 52% and 61% percent mentioned in the next few sentences? This wasn't clear to me.

***Response:*** *We acknowledge the observation made by the reviewer. We have clarified the statement to highlight the coverage of ACCESS. The statement in the text was edited to: …” ACCESS captures 59-63% of diagnosed PLHIV and 53-57% of estimated PLHIV in NSW and VIC.”*

**Comment:** I found the explanation of how the first two steps of the cascade were created to be unclear. I begins by talking about mean matching imputation but I am not clear on what is being imputed. Is this the number of people who are infected and if so, based on what data? Is it the registry data and if so, why does this need to be imputed? Or is the idea you are just imputing covariates within the registry data? It might help to start that paragraph within "In order to [state the objective] …" statement to make it clearer why the imputation was being done. And if this was based on notifications, wouldn't this be persons living with HIV who know their status and not all people with HIV?

**Response:** The Australia's National HIV Registry contains notifications with incomplete data for several key variables required for our analysis. In particular, the country of birth is missing for around 30% of notifications in the registry. Information is missing from notifications due to incomplete recording of details at the time of diagnosis, the inaccurate recording of information, and the reluctance of patients to provide accurate information due to stigma and discrimination. Missing information predominantly occurs in the first 10-15 years of Australia’s HIV epidemic but all notifications since the start of the epidemic are required to produce current estimates of the number living with HIV. To produce accurate estimates our analysis required all notifications to have complete data for the variables: year of diagnosis, age at diagnosis, sex and gender, country and region of birth, and likely exposure category. Given the missing data in some of these variables we performed statistical imputation to “fill-in” the missing values so that all notifications had complete information for each of the variables required for then analysis. The imputation approach we use is standard and uses the known data in the registry to replace missing values with statistically likely values given known values. Given the filled in data is determined statistically we produce 10 complete sets of notifications to account for potential uncertainty. We have edited this section of the manuscript to clarify this.

**Comment:** I was confused about the second 90 which appears to be limited to those in care. This doesn't seem right as the second 90 is meant to be everyone on ART and the third 90 should be everyone who is virally suppressed. Limiting to just those in care would seem to inflate the numbers and also not carry through everyone in the original denominator of 100%. I could be misunderstanding this but that is the way it read to me. The reason I think I could be wrong is the next section says "Proportion of PLDHIV retained in care on ART (2nd 90)" So if I'm wrong about the above, it isn't clear why this is the 2nd 90 as that would usually be the proportion on ART. Perhaps these are mathematically equivalent in which case I would just make that clearer.

**Response:** *Thank you for highlighting this methodological issue when developing cascades in the HIV continuum of care and it is a limitation of our study which we have now highlighted. Ideally the care cascades should be developed using a longitudinal approach, i.e. individuals diagnosed are tracked to identify ART initiation and viral suppression longitudinally. This longitudinal approach is ideal but often not practical hence the cross-sectional approach using proportions to estimate the HIV care cascade. We used two separate databases for HIV testing (Australian National HIV Registry) and clinical care (ACCESS). As highlighted, the clinical database, ACCESS, captures 59-63% of diagnosed PLHIV and 53-57% of estimated PLHIV in NSW and VIC. This made it impossible to use the traditional denominator for the 2^nd^ 90 i.e. diagnosed PLHIV hence the use of retained in care/ACCESS.*

**Comment:** The validity of the second and third 90 really depends on how representative the ACCESS database is. Can you provide any information on that given it appears to only be 50% complete?

***Response****: This has been edited and highlighted as: “ACCESS, captures 59-63% of diagnosed PLHIV and 53-57% of estimated PLHIV in NSW and VIC”.*

***** Reviewer #2:**

**Comment:** I confine my remarks to statistical aspects of this paper. The basic approach is fine, but I have some corrections and suggestions before I can recommend publication. First, I wonder why the authors didn't model the differences between groups that they seem interested in. It isn't *necessary* to do so - and the title of the paper is not about modelling - but they do seem interesting. Maybe that would be a different paper.

***Response:*** *Thank you for the suggestion to model the differences between migrants and non-migrants. We were more interested in using programmatic data to develop the cascade of care in line with UNAIDS 90-90-90 targets. However, your suggestion is welcome and we propose future analysis using a modelling approach. We have added this sentence in our discussion: “Follow-up modelling studies are required to highlight the differences between migrants and non-migrants in order to highlight additional sociodemographic characteristics that may be used to design public health programs”.*

**Comment:** Line 24, Perhaps I am confused, but this seems to contradict the result reported in the results section.

***Response:*** *The sentence made reference is: Between 2013 and 2017, the overall proportion diagnosed, on ART and with VS increased. We wanted to highlight the overall picture in NSW and VIC before stratifying the data.*

**Comment:** Line 25-28 and similarly in the results section (line 249-254) - all of these rates did increase, just not significantly. Part of this can be corrected by adding the word "significantly" but the authors should be careful not to accept the null. And, if they want to comment on statistics about differences between groups, they should model them (e.g. with a regression).

***Response:*** *Thank you for highlighting this.* *We have edited the text to indicate that there “were no significant increase”.*

**Comment:** Line 196-197 I don't understand how these plausible ranges were found or what they were used for.

***Response:*** *Plausible ranges were reported to highlight the potential uncertainty in our estimates given the uncertainty in the data and estimates used in our calculations. Details for how the plausible ranges were found are provided in each section. We have edited the text slightly to clarify this.*

**Comment:** Table 2 - I would get rid of the CIs in the cells for years.  It's not that they are wrong, but they aren't needed and they make the table very hard to read.

***Response:*** *Thank you for the suggestion, we have removed the CIs in the cells for “years”.*

***** Reviewer #3:**

**Comment:** A well written paper describing the differential treatment cascades within the Australian system, according to migrant status. Although it is not essential the authors might consider a few additions to help contextualize the paper, especially for overseas readers; Firstly, it should be noted that 28.5 % of the Australian general population is overseas born (according to the 2016 census) so the proportion of PLHIV is consistent with those figures.

***Response:*** *Thank you for highlighting this context. In 2017, 29% of Australia’s population were born overseas. This context has been added in the introduction section: “In Australia, 29% of the population were born overseas and 33% of the 27,545 estimated to be living with HIV in 2017 were born overseas mainly Southeast Asia (SEA), sub-Saharan Africa (SSA) and the Americas”.*

**Comment**: Secondly, the CD4 back projection method, to estimate the number of undiagnosed PLHIV may not be as accurate when looking at sub populations for countries over represented in late presentations (mainly due to cultural or religious stigma).

**Response**: *The reviewer is correct that stigma can result in late presentations with HIV. The CD4 back-projection method can still produce estimates even if a large proportion of an infected population are late presenters as it uses the CD4 count at diagnosis and the number of diagnoses to estimate the percentage and range in the number undiagnosed. For sub-populations with a high proportion of diagnoses occurring late then the back-projection method will estimate a higher percentage undiagnosed (reflecting the lower testing rate and late presentation) and the uncertainty will be large if the population is relatively small.*

**Comment**: Additionally, although the focus of the paper is on the apparent inequality of health outcomes for migrants, there is no separation of indigenous vs non indigenous status in the Australian born group. The poorer cascade for Australian born females suggests this may be an important issue.

***Response****: Thank you for highlighting this point. As acknowledged, the aim of this paper was to investigate the HIV cascade among people with migrant and non-migrant backgrounds in Australia. The HIV cascade among indigenous Australians is also important however this population face different barriers to testing and treatment that are beyond the scope of this paper. The poorer cascade among Australian-born females calls for comparisons between indigenous and non-indigenous Australians which has been done in the HIV surveillance annual reports. We have reflected this in the discussion.*

**Comment**: Finally, the assumption that overseas born PLHIV is a surrogate for inability to access care is a bit of an over simplification. Many overseas born people do have PR or residency and can access Medicare as well as anyone else. For those without Medicare, all sexual health clinics provide free care and can access free antiviral medication now. It would seem that the key barrier is reluctance of the migrant to access testing or care, rather than these services are not available to them. this is reflected in the increasing rates of on therapy and VS in all groups but not in any improvement in the proportion diagnosed.

***Response****: Indeed, using location of birth is an over simplification of Medicare eligibility. This has been highlighted in the limitations section as: “…the analysis considered country of birth as a proxy for migrant status and Medicare eligibility, and may have misclassified people born in other countries who were Australian citizens and Medicare eligible”. Direct Medicare eligibility data was not collected and would be required in future studies including a higher completion rate of country of birth and year of arrival. We acknowledge that while many migrants may be Medicare eligible, the eligibility may not be a guarantee that an individual will seek HIV testing. Even if services are available, it doesn’t necessarily mean that people know about them. We have also mentioned that stigma is major barrier. People may have also faced racism and discrimination within healthcare settings despite being Medicare eligible.*
