# Supplementary material for: Gaps in the HIV diagnosis and care cascade for migrants in Australia, 2013–2017: A cross-sectional study
Source: PLoS Med. 2020 Mar 10;17(3):e1003044. doi: 10.1371/journal.pmed.1003044 (PMC7064172; doi:10.1371/journal.pmed.1003044)
Supplement: Attachment — Submitted filename: Responses to reviewers.docx [file pmed.1003044.s006.docx]

**RESPONSES TO REVIEWERS’ COMMENTS AND EDITOR’S REQUESTS**

**Requests from Editors:**

**Comment:** Please adapt the title slightly: we suggest "Gaps in the HIV diagnosis and care cascade for migrants in Australia, 2013-2017: a cross-sectional study".

***Response:*** *Thank you for the suggestion. The edit has been applied on the title.*

**Comment:** Please revise your abstract, aiming to improve readability of the "methods and findings" subsection.

***Response:*** *The methods and findings subsection were edited to improve readability.*

**Comment:** At line 4 in your abstract, we suggest adding a few words to quote the UNAIDS targets.

***Response:*** *Thank you, we added the sentence: “Fast-Track targets are aligned to the HIV diagnosis and care cascade and entails achieving 90-90-90 (90% of people living with HIV diagnosed, 90% of diagnosed on treatment, and 90% of those on treatment achieving viral suppression) by 2020 and 95-95-95 by 2030”*

**Comment:** At line 24 (and line 246 in the main text), please quote the number or proportion of males, say, in the total cohort.

***Response:*** *We added the proportion of males in the total cohort of estimated PLHIV and changed the wording to: “We found that in 2017, 17,760 were estimated PLHIV in NSW and VIC and 90% of them were males.”*

**Comment:** To the final sentence of the "methods and findings" subsection of your abstract, please add 1-2 further limitations. We ask you to include the point made at line 379, i.e., that estimation of the "second 90" was done by assessing those in care.

***Response:*** *Thank you for the suggestion. We have added the following limitations: “Additionally, we used a cross-sectional instead of a longitudinal study design to develop the cascades and used the number retained as opposed to using all individuals diagnosed to calculate the proportions on ART.”*

**Comment:** Please restructure the "author summary" so that it consists of the existing three sections, but with each section comprising 3-4 bulleted points. The individual points should consist of one sentence, in general. Please consult one or two recent research papers published in PLOS Medicine to get a sense of the preferred style.

***Response:*** *The author summary was edited as follows:*

***Why was this study done?***

- *Reaching the end of the AIDS epidemic by achieving the UNAIDS Fast-Track targets requires tracking progress across all population groups.*
- *Fast-Track targets entails reaching 90-90-90 (90% of people living with HIV diagnosed, 90% of diagnosed on treatment, and 90% of those on treatment with viral suppression) by 2020, and 95-95-95 by 2030.*
- *Migrants often face barriers to health care access due to their migration status, sociocultural influence, financial constraints, and stigma.*
- *There is limited data on HIV diagnosis, care and treatment among migrants which is required in order to streamline the HIV response.*

***What did the researchers do and find?***

- *We developed HIV diagnosis and care cascades for migrants and non-migrants in Australia between 2013-2017.*
- *The overall cascade was high in New South Wales and Victoria (i.e. 90-90-95).*
- *Migrants had inferior cascades (85-85-93) compared to non-migrants (94-90-96) including migrants reporting male-to-male HIV exposure (84-83-93) compared to non-migrants (96-92-96).*
- *Between 2013 and 2017, subpopulation cascades improved except for females irrespective of migration status and migrants from Southeast Asia.*

***What do these findings mean?***

- *In our study, while the overall HIV diagnosis and care cascades improved over time in Australia, migrants are lagging behind.*
- *Failure to develop subpopulation cascades may lead to gaps going un-noticed.*
- *To address gaps, there is need for streamlined strategies especially among migrant populations to ensure timely HIV testing and linkage to care.*

**Comment:** At line 254 and elsewhere in the ms, please substitute "(Table 1)". In general, square brackets should only be used inside parentheses.

***Response:*** *Thank you for highlighting this. We have substituted the square brackets throughout the manuscript.*

**Comment:** At line 291, please remove "by 6%" (which is implicit in the point estimate) to leave "... increased (IRR 1.06 ... )".

***Response:*** *Thank you for identifying this duplication. This wording has been edited out as suggested.*

**Comment:** Please add initial zeroes to the p values in table 2.

***Response:*** *Table 2 has been edited to ensure that all p-values have the initial “0” where appropriate.*

**Comment:** At line 358, should that be "surveillance data"?

***Response:*** *Thank you for identifying this typo. The correction has been made.*

**Comment:** Throughout the text, please adapt the formatting of reference callouts as follows: "... risk of transmission [36,37].".

***Response:*** *The appropriate formatting of multiple reference callouts has been rectified throughout the manuscript.*

**Comment:** In the reference list, please ensure that journal names are abbreviated as appropriate (e.g., reference 4).

***Response:*** *We have made changes to the reference list ensuring that the journal abbreviations are reflected correctly.*

**Comment:** Please remove ampersands from the reference list (e.g., reference 10).

***Response:*** *Thank you. The correction has been made.*

**Comment:** Please correct the spelling of the journal name for reference 15.

***Response:*** *The journal name typo has been corrected.*

**Comment:** To the attached STROBE checklist, please add paragraph numbers, where applicable, to the rightmost column.

**Response:** The STROBE checklist was updated to include paragraph numbers.

**Comments from Reviewers:**

***** Reviewer #2:**

**Comment:** The authors have addressed my concerns and I now recommend publication
